# Cross-modal attentional effects of rhythmic sensory stimulation

**DOI:** 10.3758/s13414-022-02611-2

**Published:** 2022-11-16

**Authors:** Ulrich Pomper, Bence Szaszkó, Simon Pfister, Ulrich Ansorge

**Affiliations:** 1 Department of Cognition, Emotion, and Methods in Psychology, Faculty of Psychology, University of Vienna, Liebiggasse 5, 1010 Vienna, Austria; 2 Vienna Cognitive Science Research Hub, University of Vienna, Vienna, Austria; 3 Research Platform Mediatised Lifeworlds, University of Vienna, Vienna, Austria

**Keywords:** Cross-modal, Entrainment, Oscillations, Theta, Auditory, Visual

## Abstract

Temporal regularities are ubiquitous in our environment. The theory of entrainment posits that the brain can utilize these regularities by synchronizing neural activity with external events, thereby, aligning moments of high neural excitability with expected upcoming stimuli and facilitating perception. Despite numerous accounts reporting entrainment of behavioural and electrophysiological measures, evidence regarding this phenomenon remains mixed, with several recent studies having failed to provide confirmatory evidence. Notably, it is currently unclear whether and for how long the effects of entrainment can persist beyond their initiating stimulus, and whether they remain restricted to the stimulated sensory modality or can cross over to other modalities. Here, we set out to answer these questions by presenting participants with either visual or auditory rhythmic sensory stimulation, followed by a visual or auditory target at six possible time points, either in-phase or out-of-phase relative to the initial stimulus train. Unexpectedly, but in line with several recent studies, we observed no evidence for cyclic fluctuations in performance, despite our design being highly similar to those used in previous demonstrations of sensory entrainment. However, our data revealed a temporally less specific attentional effect, via cross-modally facilitated performance following auditory compared with visual rhythmic stimulation. In addition to a potentially higher salience of auditory rhythms, this could indicate an effect on oscillatory 3-Hz amplitude, resulting in facilitated cognitive control and attention. In summary, our study further challenges the generality of periodic behavioural modulation associated with sensory entrainment, while demonstrating a modality-independent attention effect following auditory rhythmic stimulation.

## Introduction

Sensory signals are abundant with temporal regularities, stemming both from external sources, like animal movements or vocalizations, as well as from cyclic patterns in exploratory behaviours, such as saccades, sniffing, or whisking (Ding et al., 2017; Kotz et al., 2018; Morillon et al., 2015; Schroeder et al., 2010). Likewise, neural activity is characterized by rhythmic patterns, most prominently as oscillations in the range of 1 to 100 Hz (Buzsáki, 2006; Wang et al., 2010).

Recently, the concept of neural entrainment has gained increasing popularity, which suggests that cortical oscillations can synchronize with temporal regularities in incoming sensory information (Haegens & Zion Golumbic, 2018; Lakatos et al., 2019; Obleser & Kayser, 2019; Rimmele et al., 2018). As ongoing neural oscillations reflect fluctuations in excitability, their entrainment to a regular sensory input aligns phases of high (or low) processing capabilities with moments of incoming information, thereby selectively enhancing (or suppressing) it (Hickok et al., 2015; Lawrance et al., 2014; Spaak et al., 2014).

For example, Hickok et al. (2015) presented participants with 3 s of 3 Hz amplitude- modulated noise as an entrainment stimulus, followed by a faint auditory target at various stimulus onset asynchronies (SOAs). The authors observed a fluctuation in target detection performance at roughly 3 Hz, with facilitated performance at SOAs out of phase with the preceding noise. This suggests that the temporal allocation of attention gets entrained by the regularly oscillating stimuli, presumably via phase alignment of neural oscillations. Indeed, several studies have observed concomitant modulations of neural activity measured via electroencephalography (EEG) or magnetoencephalography (MEG), particularly at the frequency of the entrainment stimulus (Bauer et al., 2021; de Graaf et al., 2013; Mathewson et al., 2012; Spaak et al., 2014). Spaak et al. (2014), for instance, presented 10 Hz rhythmic flashes to one visual hemifield and arrhythmic flashes to the other, while recording EEG from human participants. They observed an increase in both alpha (8–12 Hz) power and intertrial phase coherence, specifically contralateral to the rhythmic stimulation. This alpha modulation continued beyond the offset of the stimulation and led to a cyclic modulation of subsequent psychophysical performance, with better performance for visual targets presented out-of-phase with the preceding flashes. Notably, the concept of entrainment is closely related to dynamic attending theory (Jones, 1976; Jones et al., 2002), which states that attention can be deployed dynamically to moments at which important events are predicted to occur, and, in the auditory modality, is a candidate mechanism for extracting syllabic information from continuous speech (Ding & Simon, 2014; Kösem et al., 2018; Zoefel & VanRullen, 2015).

So far, behaviourally relevant effects of entrainment have been observed in both the visual (e.g., de Graaf et al., 2013; Mathewson et al., 2012; Spaak et al., 2014) and auditory (e.g., Henry et al., 2014; Hickok et al., 2015; Lawrance et al., 2014) modalities. However, the extent to which entrainment effects are restricted to the stimulated sensory modality or can cross over^1^ to other modalities is currently not well understood. While Spaak et al. (2014) observed only hemisphere-specific effects of rhythmic visual stimulation on neural activity in visual cortex and on behaviour, suggesting little spread of effects to other sensory modalities, a number of studies have found a unidirectional crossing of entrainment effects from the auditory to the visual (Barnhart et al., 2018; Bauer et al., 2021) and from the visual to the auditory (Albouy et al., 2022; Biau et al., 2021; Crosse et al., 2015) domain. Importantly, a potential bidirectional spread of entrainment effects between the auditory and visual domains within the same experimental paradigm and participants has not yet been investigated.

Additionally, while sensory entrainment has been reported numerous times using various different experimental protocols, evidence regarding this phenomenon remains mixed. Particularly, several recent studies have failed to directly replicate previous findings or produce similar effects (de Graaf & Duecker, 2021; Duecker et al., 2021; Lin et al., 2021; Sun et al., 2021). Likewise, conflicting results have been obtained for some of the key elements of entrainment. For instance, facilitated stimulus processing has been observed at time points in-phase with the preceding regular stimulus by some studies (de Graaf et al., 2013; Lawrance et al., 2014; Mathewson et al., 2012) and out of phase by others (Barnes & Johnston, 2010; Hickok et al., 2015; Spaak et al., 2014). A likely reason is that the exact necessary side conditions of entrainment effects are not yet known, meaning that prior demonstrations are often instances of discovery-oriented research that cannot be logically derived from formal theory (as in theory-testing research), but are rather based on general arguments for the possibility of entrainment and, as such, have a lower chance of successful replication (cf. Oberauer & Lewandowsky, 2019).

Finally, irrespective of their phase, it is unclear under which circumstances entrainment effects can continue beyond the offset of the entrainment stimulus, and for how long (cf. Capilla et al., 2011; Hickok et al., 2015; for a discussion see Haegens & Zion Golumbic, 2018).

Taken together, these inconsistencies highlight the need for further investigations into the necessary preconditions as well as potential behavioural consequences of rhythmic sensory stimulation.

In the present study, we aimed at addressing these current issues by varying pretarget stimulation modality, target modality, and target presentation time in a full-factorial design. Participants were first presented with 3 s of either visual or auditory rhythmic stimulation,^2^ followed by a visual or auditory target at six possible time points after stimulation offset, either in-phase or out-of-phase relative to the stimulus.

Following the previous evidence reviewed above, we hypothesized that (1) by staying close to previously used experimental protocols (particularly Hickok et al., 2015), we can replicate the phase effects of rhythmic stimulation and observe facilitated behavioural performance out-of-phase compared with in-phase with the preceding stimulus; (2) rhythmic stimulation effects can cross from the visual to the auditory modality and vice versa; and (3) rhythmic stimulation effects can persist following the offset of the inducing sensory input, but diminish over time. While Hypothesis (3) could be regarded a logical (but general and, therefore, relatively trivial) necessity of any stimulus-specific perceptual processing and, thus, as a case of theory-testing research, Hypotheses (1) and (2) are instances of discovery-oriented research, as these hypotheses are formulated mainly in analogy to related observations in different experimental protocols (cf. Oberauer & Lewandovsky, 2019).

To anticipate the results, we observed a general, phase-independent effect of rhythmic stimulation on performance, which was more pronounced following auditory than visual rhythmic stimulation. However, our data showed no evidence of periodic fluctuations in performance following rhythmic stimulation, thus providing further evidence against the generality of this phenomenon.

## Methods

### Participants

Twenty-six psychology students from the University of Vienna participated in the experiment in exchange for partial course credit or for a financial compensation of 10 EUR per session. The present sample size was set based on previous studies on sensory entrainment, many of which have included between 20 and 30 participants (e.g., Bauer et al., 2021; Duecker et al., 2021; Lawrance et al., 2014; Miller et al., 2013; Spaak et al., 2014; Sun et al., 2021). We excluded five participants due to a false.-alarm rate exceeding 25%, leaving us with a sample of 21 participants (13 female, *M*_age_ = 24.1 years , *SD*_age_ = 3.13 years, ranging from 20 to 30 years). All participants reported or had normal or corrected-to-normal visual auditory acuity. Participants provided written consent prior to the experiment and were treated according to the Declaration of Helsinki. We further followed the Austrian Universities Act, 2002 (UG2002, Article 30 § 1), which states that only medical universities or researchers conducting applied medical research are required to obtain an additional approval by an ethics committee. Therefore, no additional ethical approval was required for our study.

### Apparatus

We conducted the experiment in a dimly lit room. Stimuli were presented on a 24.5-in. G2590PX AOC Gaming LCD monitor (visible part of the display: 54.4 cm × 30.3 cm) with a resolution of 1,920 × 1,080 pixels and a refresh rate of 60 Hz. A constant viewing distance of 57 cm was ensured by a chin rest. The experiment was programmed with and executed by OpenSesame (Version 3.1; Mathôt et al., 2012). For the auditory stimuli, Panasonic RP-HT 265 headphones were used and the volume was adjusted to a subjectively pleasant level for each subject and kept constant during the experimental session.

### Stimuli and procedure

The experiment consisted of a total of 840 trials and was divided into two parts (420 trials per session). While in one of the sessions participants received rhythmic visual stimulation at the beginning of each trial (in the following: *visual pretarget stimulation trials*), in the other session, they received rhythmic auditory stimulation at trial beginning (*auditory pretarget stimulation trials*). The order of the sessions was counterbalanced across participants. Opportunities for self-paced breaks were given after every 28 trials, resulting in 15 blocks per session.

**Fig. 1:**
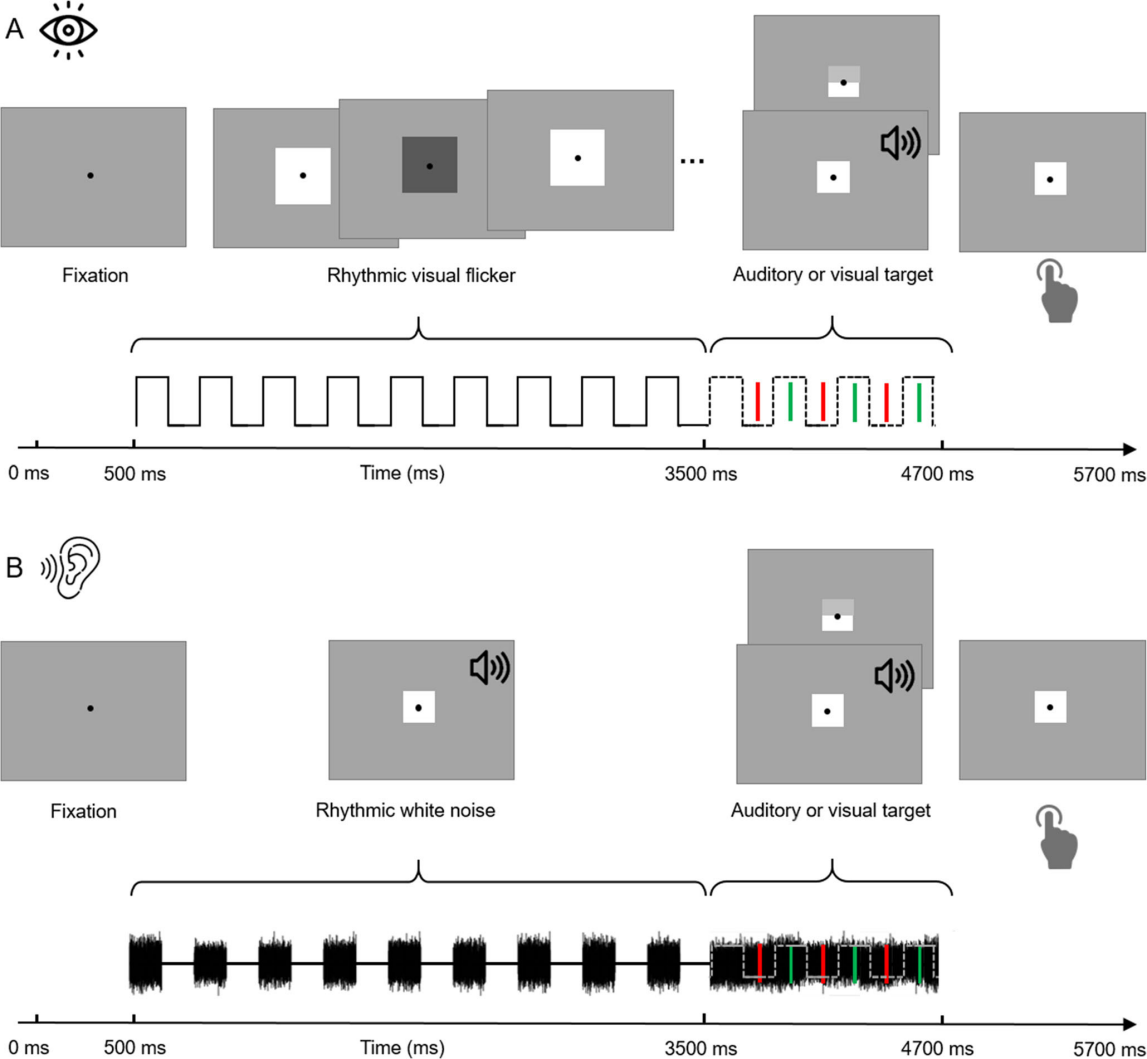
Trial design. **a** Visual pretarget stimulation condition. After an initial fixation period of 500 ms, a rectangle flickering rhythmically at 3 Hz was presented for 3 s. Subsequently, a visual or auditory target (or no target, not shown) was presented at one of six possible time points, either in-phase (green bars) or out-of-phase (red bars) with the preceding rhythmic stimulus. Participants reported the location of the visual target (top or bottom) or the pitch of the auditory target (high or low) via a speeded button press. The continuous waveform illustrates the rhythmic stimulus. The dotted waveform illustrates the expected course of the stimulus if it had been continued. Additionally, continuous white noise was auditorily presented between the offset of the fixation display and the end of the trial. **b** Auditory pretarget stimulation condition. Identical to (**a**), except that following fixation, instead of the visual flicker, a 3-Hz amplitude-modulated white noise sound was presented for 3 s. After 3 s, the white noise was presented continuously until the end of the trial

Visual stimuli were presented on a grey background (luminance: 14.7 cd/m^2^), with a black fixation dot (radius: 0,3°) at screen centre, present throughout the experiment. Figure 1 shows one exemplary trial. Each trial was 5.7-s long and began with a fixation interval of 500 ms, during which only the black fixation dot was shown. Following the fixation interval, in auditory pretarget stimulation trials, a 3-s long rhythmic white noise , amplitude modulated at 3 Hz (modulation depth: 100%; rise-and-decay time 10 ms) was presented. We chose the modulation frequency of 3 Hz (equalling a cycle length of 333 ms) analogously to the study by Hickok et al. (2015), as well as because it is in the range of numerous naturally occurring auditory stimuli, like human speech (Chandrasekaran et al., 2009; Elliott & Theunissen, 2009; Luc et al., 2012; Poeppel, 2003). In the visual domain, likewise, studies have shown behaviourally relevant entrainment effects following rhythmic stimulation in the theta frequency range (Bertrand et al., 2018; Köster, Langeloh, et al., 2019a; Köster, Martens, et al., 2019b). This makes a stimulation frequency of 3 Hz well suited for the present full factorial design, which requires equal frequencies for testing both within and across modality effects. During rhythmic auditory pretarget stimulation, a white square (side length: 1°) was presented at screen centre. In contrast, in visual pretarget stimulation trials, a 3-s long rhythmic 3-Hz flicker of a square with 4° side length at screen centre changed colours between white (17.3 cd/m^2^) and dark grey (10.2 cd/m^2^). During rhythmic visual pretarget presentation, continuous auditory white noise was presented. After the rhythmic pretarget stimulation, in both auditory and visual pretarget stimulation trials, continuous white noise and the white square (the same as during auditory pretarget stimulation) were presented for 1,200 ms. This period served as a target window, during which a potential auditory or visual target could appear for 50 ms. Participants had 1 s to press a button to indicate target onset.

Auditory and visual targets could appear at six distinct points in time during each trial. Three of these were chosen to be in-phase with the rhythmic pretarget stimulus (417 ms / 25 frames, 750 ms / 45 frames, and 1,083 ms / 65 frames after the offset of the entraining stimulus), and the other three out-of-phase (250 ms / 15 frames; 583 ms / 35 frames; 917 ms / 55 frames). In other words, if the rhythmic stimulus would have continued, in-phase targets would have appeared exactly halfway through presentation of a noise burst or a dark grey visual square, while out-of-phase targets would have appeared exactly halfway through the silent gap between noise bursts or during the white visual square. By using six time points, we could investigate the time course of potential rhythmic stimulation effects after rhythmic stimulation has ended. Target modalities were pseudorandomized across trials within each block, with 12 out of the 28 trials containing a visual target, 12 trials containing an auditory target, while four of the 28 trials served as catch trials, containing no target.

Two distinct stimuli served as auditory targets: a 660 Hz sinusoidal pure tone (in the following: low tone), and an 880 Hz sinusoidal pure tone (high tone), embedded within the continuous white noise, both of 50 ms duration with a 2-ms rise-and-decay time. In case of an auditory target, participants had to press the “up arrow” button in response to a high tone, and the “down arrow” button in response to a low tone. Analogously, the visual targets consisted of a 50 ms long change of colour from white to grey in the upper half or lower half of the white square. Participants had to press “up arrow” if the upper part of the white rectangle flickered, and “down arrow” if the lower half flickered.

After each block, participants received additional written feedback on their mean response time (RT), hit rate, and false-alarm rate in the previous block. Prior to the experiment, individual thresholds for the perception of the visual and auditory stimuli were assessed through an up-and-down staircase procedure. Stimulus intensities (i.e., loudness and contrast of the auditory and visual targets, respectively) were then continuously adapted throughout the experiment (after every fourth trial) to yield 75% hit rate. To familiarize participants with the two different auditory target tones, both were presented prior to the threshold determination procedure. After threshold assessment, participants continued with 14 practice trials before proceeding to the main experiment. The experimental instructions stressed both speed and accuracy of the responses, as well as the necessity to minimize the false-alarm rate.

### Data analysis

We used RStudio (Version 1.4.1717; RStudio Team, 2020) with R (Version 4.1.2; R Core Team, 2021) and the R packages broom (Version 0.7.9; Robinson et al., 2021), data.table (Version 1.14.2; Dowle & Srinivasan, 2020), dplyr (Version 1.0.7; Wickham et al., 2021), effsize (Version 0.8.1; Torchiano, 2020), ggplot2 (Version 3.3.5; Wickham, 2016), ggrepel (Version 0.9.1; Slowikowski, 2021), MBESS (Version 4.8.1; Kelley, 2021), and schoRsch (Pfister & Janczyk, 2016) for data analyses.

We excluded participants with a false-alarm rate higher than 25% (*N* = 5). We only included RTs from trials with correct responses into our analyses and excluded trials with RTs more than 2.5 standard deviations above or below the condition’s mean RTs (*M* = 11.9, *SD* = 3.1). For both of our two dependent variables, RT and hit rates (HR), we conducted a 2 × 2 × 2 × 3 repeated-measures analysis of variance (ANOVA), with the independent variables stimulation modality (auditory, visual), target modality (auditory, visual), phase (in-phase, out-of-phase), and time (T1, T2, T3). Significant interactions were followed up by *t* tests.

For the ANOVAs, we used partial eta-squared (${\upeta}_{\mathrm{p}}^2$) as effect size measure (Richardson, 2011). For all further analyses, we used Cohen’s *d* as effect size (standardized with the standard deviation of the effect in question) and applied Hedges’s correction factor (Hedges, 1981). We used a significance level of α = .05 and Holm-corrected *p* values for multiple comparisons (Holm, 1979).

## Results

### Hit rates

**Fig. 2:**
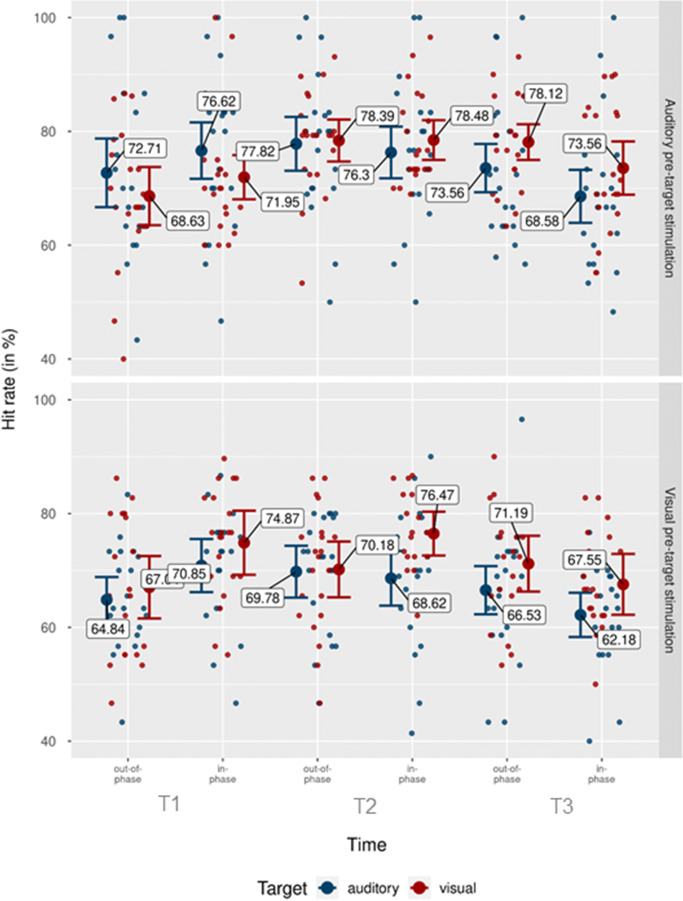
Hit rates by pretarget stimulation modality, target modality, time point, and phase. Mean of participants’ hit rates for auditory (top) and visual (bottom) pretarget stimulation modalities. Blue dots indicate mean hit rates for the auditory target condition. Red dots show mean hit rates for the visual target condition. Individual data points represent mean hit rate per subject and condition. Error bars show 95% CI. (Colour figure online)

**Fig. 3:**
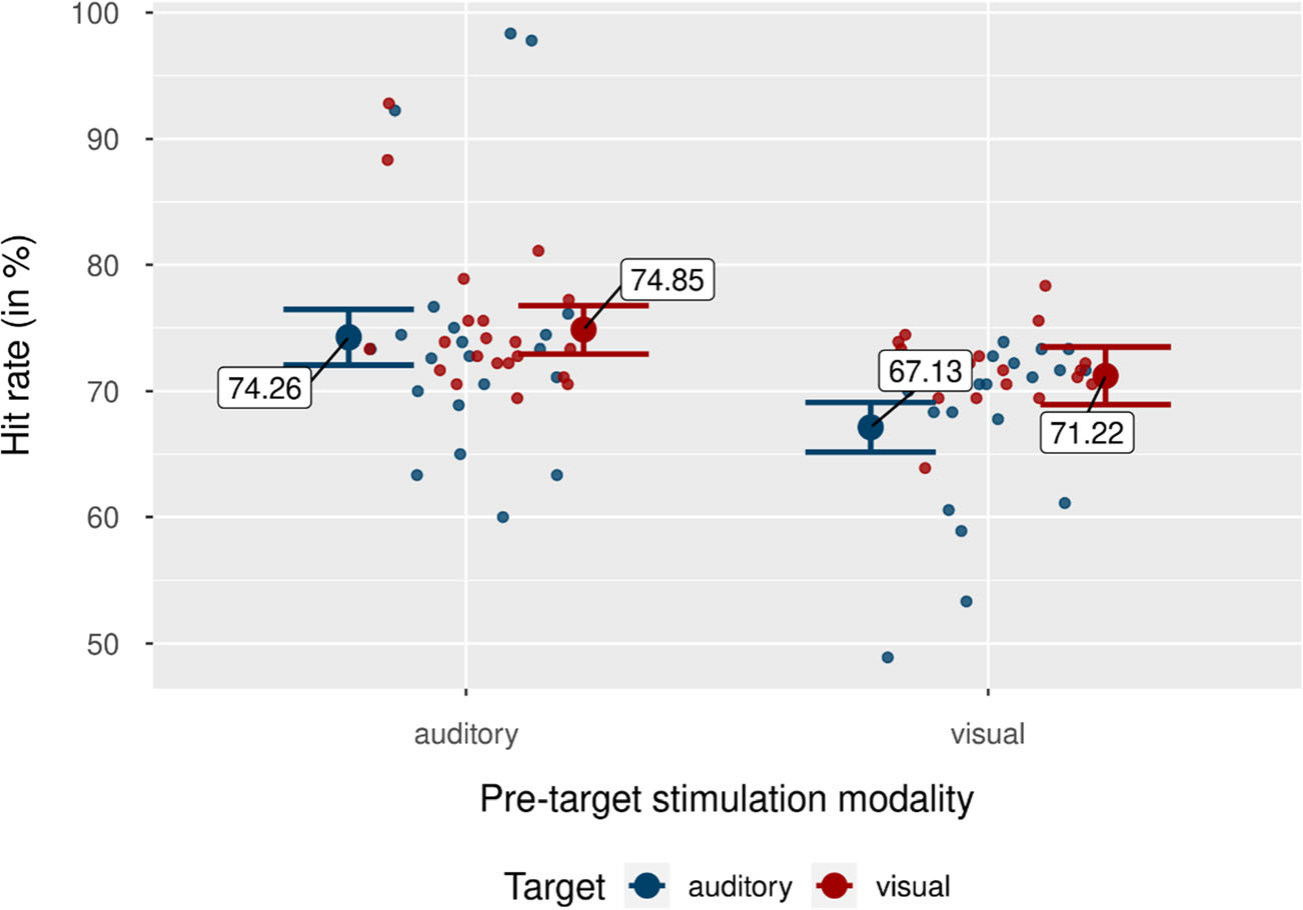
Hit rates by pretarget stimulation modality and target modality. Mean of participants’ hit rates for auditory and visual target modalities. Blue dots indicate mean hit rates for the auditory target condition. Red dots show mean hit rates for the visual target condition. Individual data points represent mean hit rate per participant and condition. Error bars show 95% CI. (Colour figure online)

To assess the impact of rhythmic stimulation on participants’ accuracy, we conducted a 2 × 2 × 2 × 3 repeated-measures ANOVA, with pretarget stimulation modality (auditory, visual), target modality (auditory, visual), phase (in-phase, out-of-phase), and time (T1, T2, T3) as independent variables, and hit rates (HR) as dependent variable. Figure 2 shows individual and grand mean hit rates separately for each condition. We found a main effect of pretarget stimulation modality, *F*(1, 20) = 11.26, *p* = .003, ${\upeta}_{\mathrm{p}}^2$ = .36, with higher hit rates in auditory pretarget stimulation trials, *M* = 74.56%, than in visual pretarget stimulation trials, *M* = 69.18%. We also found a main effect of time, *F*(2, 40) = 7.43, *p* = .002, ${\upeta}_{\mathrm{p}}^2$ = .27, with the highest hit rate in T2, *M* = 74.50%, followed by T1, *M* = 70.94%, and T3, *M* = 70.16%. Further, the interaction between pretarget stimulation modality and target modality was significant, *F*(2, 40) = 4.93, *p* = .038, ${\upeta}_{\mathrm{p}}^2$ = .20. This interaction was mainly driven by a more homogenous effect of auditory pretarget stimulation on both visual and auditory hit rate, compared with visual pretarget stimulation, visible in Fig. 3. Following auditory pretarget stimulation, performance for both target modalities was highly similar, with a slight numerical but non-significant performance advantage towards visual targets, *M*_Diff_ *=* 0.59%, *SD* = 6.90%, 95% CI [−2.55, 3.73], *t*(20) = 0.39, *p* = .700, *d*_*unb*_ = 0.08 [−3.44, 0.51]. Following visual pretarget stimulation, performance in visual target trials was significantly better than in auditory target trials, *M*_Diff_ = 4.08%, *SD* = 7.86%, 95% CI [0.50, 7.66], *t*(20) = 2.38, *p* = .028, *d*_*unb*_ = 0.50 [0.06, 0.97].

**Fig. 4:**
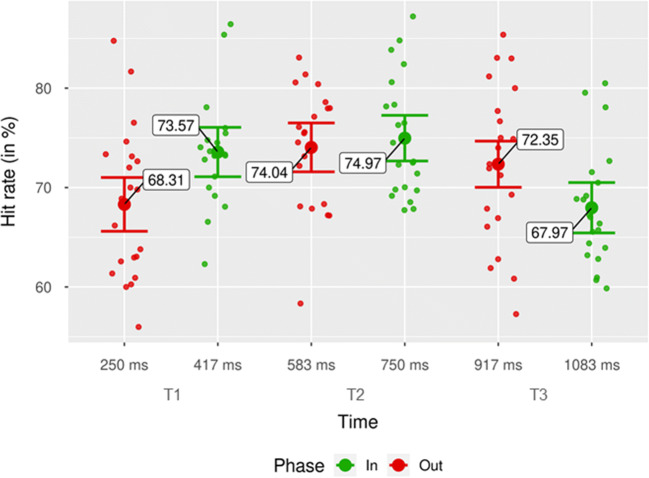
Hit rates by phase for each time point. Mean of participants’ hit rates at each time point (time in ms between pretarget stimulation offset and target appearance). Green dots indicate mean hit rates for the in-phase condition. Red dots show mean hit rates for the out-of-phase condition. Individual data points represent mean hit rate per participant and condition. Error bars show 95% CI. (Color figure online)

We also found a significant interaction between phase and time, *F*(2, 40) = 9.57, *p* < .001, ${\upeta}_{\mathrm{p}}^2$ = .32. The interaction was driven by two main factors (Fig. 4): first, by lower hit rates in out-of-phase trials than in in-phase trials at T1, *M* = −5.26%, 95% CI [−8.23, −2.30, ], *SD* = −6.52%, *t*(20) = −3.70, *p =* .001*, d*_*unb*_ = −0.78 [−1.30, −0.31]; second, by higher hit rates in out-of-phase trials than in in-phase trials at T3, *M* = 4.38%, 95% CI [0.94, 7.82], *SD* = 7.56%, *t*(20) = 2.65, *p* = .015, *d*_*unb*_ = 0.56 [0.11, 1.04]. The difference in hit rates between in-phase and out-of-phase trials at T2 was not significant, *M* = −0.93%, 95% CI [−3.60, 1.75], *SD* = 5.88%, *t*(20) =−0.67, *p* = .479, *d*_*unb*_ = −0.15 [−0.59, 0.27].

**Fig. 5:**
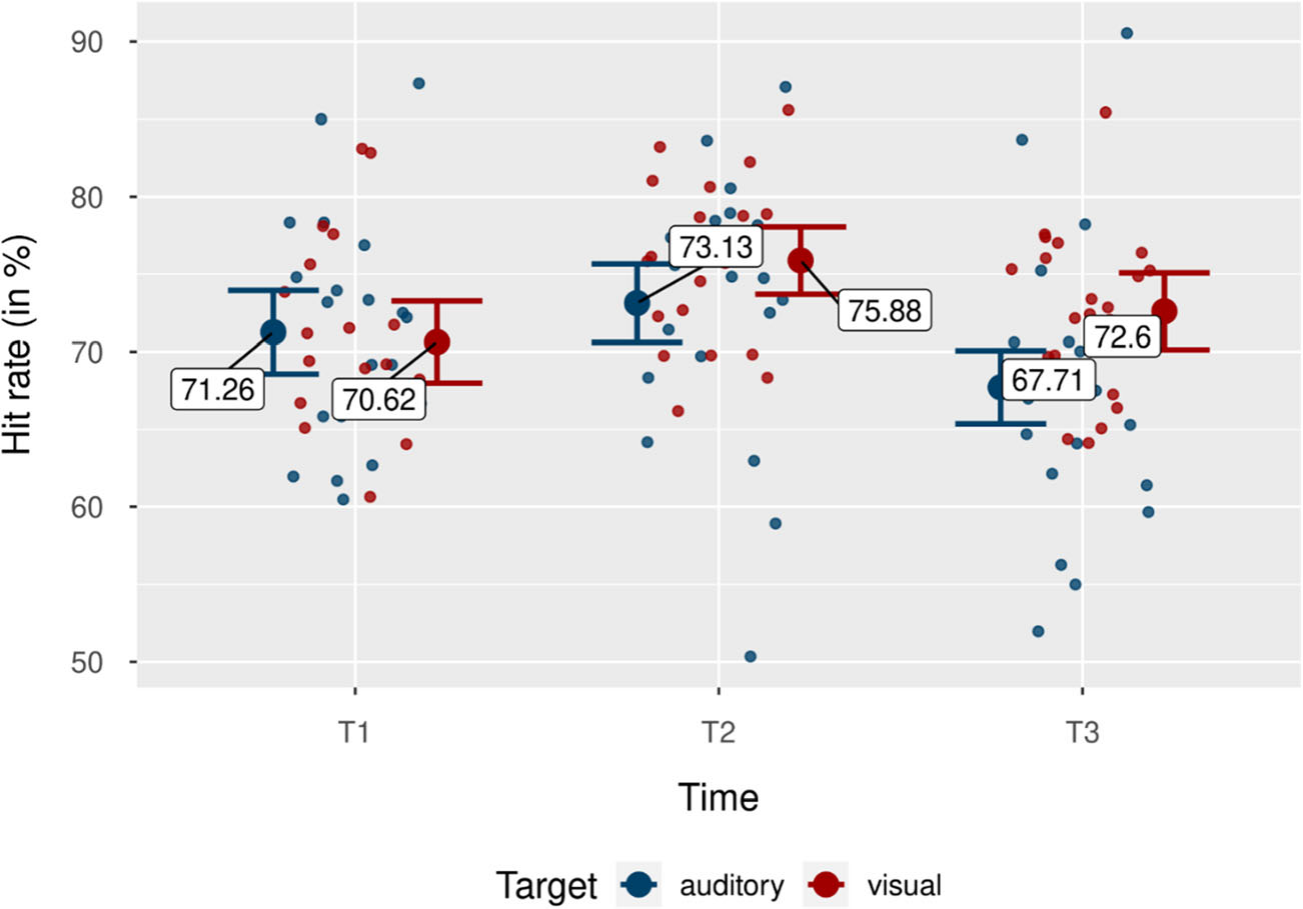
Hit rates by target modality for each time point. Blue indicate mean hit rates for the auditory target condition, red dots show mean hit rates for the visual target condition. Individual data points represent mean hit rate per subject and condition. Error bars show 95% CI. (Color figure online)

The interaction between target modality and time was significant as well, *F*(2, 40) = 3.29, *p* = .048, ${\upeta}_{\mathrm{p}}^2$ = .14 (see Fig. 5). This effect was due to performance differences between modalities increasing over time, with T3 having the greatest hit-rate difference between trials with visual and auditory targets, *M* = 4.89%, *SD* = 9.25%, 95% CI [0.68, 9.10], *t*(20) = 2.42, *p* = .025, *d*_*unb*_ = 0.51 [0.07, 0.98]. Differences between visual and auditory performance at T1 and T2 were not significant (both *p*s > .196). All other main effects and interactions were also not significant (all *p*s > .086).

### Response times

**Fig. 6:**
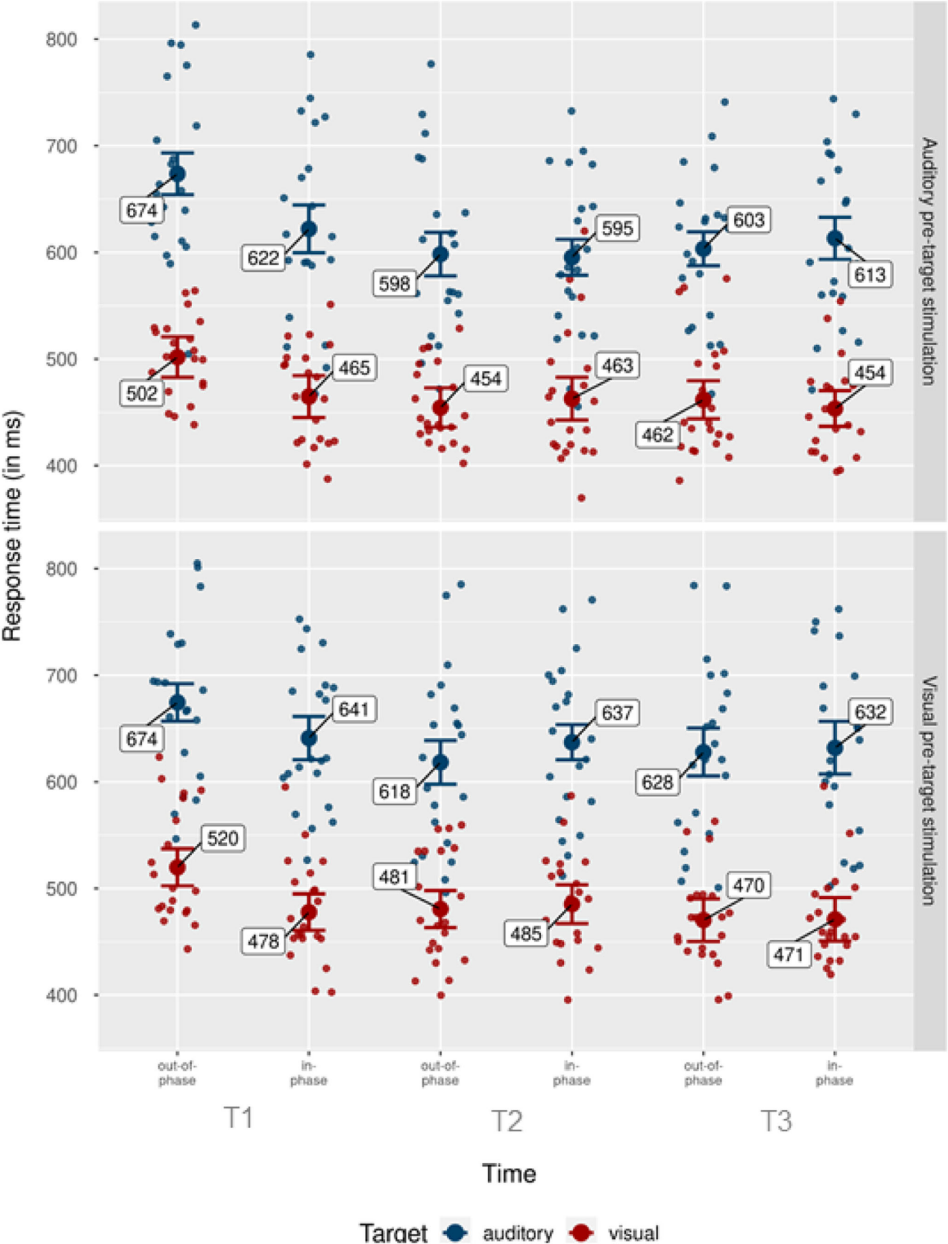
Response times by pretarget stimulation modality, target modality, time point, and phase. Mean of participants’ correct response times for auditory (top) and visual (bottom) pretarget stimulation modalities. Blue dots indicate mean RTs for the auditory target condition. Red dots show mean RTs for the visual target condition. Individual data points show average hit rate per participant and condition. Error bars show 95% CI. (Colour figure online)

Analogously to the hit rate analysis, we conducted a 2 × 2 × 2 × 3 repeated-measures ANOVA, with the independent variables pretarget stimulation modality (auditory, visual), target modality (auditory, visual), phase (in-phase, out-of-phase), and time (T1, T2, T3). Figure 6 shows individual and grand mean RTs separately for each condition. We found a significant main effect of pretarget stimulation modality, *F*(1, 20) = 9.28, *p* = .006, ${\upeta}_{\mathrm{p}}^2$ = .32, due to faster response times in trials with auditory pretarget stimulation, *M* = 542 ms, than in visual pretarget stimulation, *M* = 561 ms. We also found a main effect of target modality, *F*(1, 20) = 197.50, *p* < .001, ${\upeta}_{\mathrm{p}}^2$ = .91, driven by significantly faster response times in trials with visual targets, *M* = 475 ms, compared with trials with auditory targets, *M* = 628 ms. There was also a main effect of phase, *F*(1, 20) = 17.54 *p* < .001, ${\upeta}_{\mathrm{p}}^2$ = .47, due to faster responses in trials in which the target was presented in-phase, *M* = 546 ms, compared with out-of-phase, *M* = 557 ms. Finally, there was a main effect of time, *F*(1, 40) = 36.48, *p* < .001, ${\upeta}_{\mathrm{p}}^2$ = .65, with trials in T3 (latest time point after pretarget stimulation), and T2 showing faster responses, *M* = 542 ms and *M* = 545 ms, respectively, and T1 being slower, *M* = 572 ms.

**Fig. 7:**
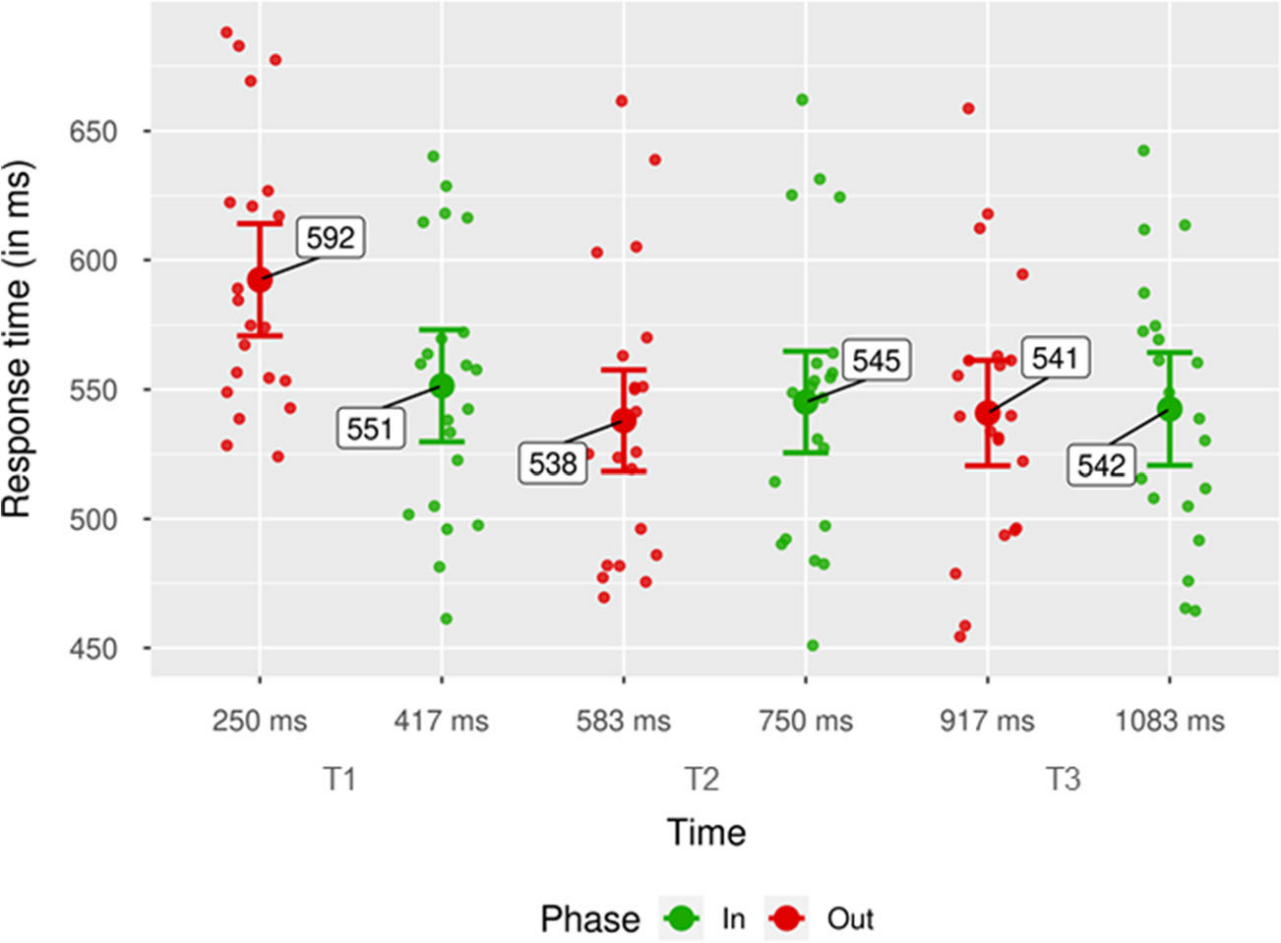
Response times by phase for each time point. Mean of participants’ correct response times at each time point (time in ms between pretarget stimulation offset and target appearance). Green dots indicate mean hit rates for the in-phase condition. Red dots show mean hit rates for the out-of-phase condition. Individual data points represent mean hit rate per subject and condition. Error bars show 95% CI. (Colour figure online)

Further, the interaction between phase and time was significant, *F*(2, 40) = 33.47, *p* < .001, ${\upeta}_{\mathrm{p}}^2$ = .63. Figure 7 shows the respective RTs per time point and phase. The interaction was mainly driven by faster responses in in-phase trials than out-of-phase trials at T1, *M* = 41 ms, 95% CI [30, 52], *SD* = 25 ms, *t*(40) = 7.61, *p* < .001, *d*_*unb*_= 1.60 [1.04, 2.40]. The difference between in- and out-of-phase trials at T2 was marginally significant, *M* = −7 ms, 95% CI [−16, 1], *SD* = 18 ms, *t*(40) = −1.79, *p* = .088, *d*_*unb*_ = −0.38 [−0.83, 0.06], while the difference at T3 was not, *M* = −2 ms, 95% CI [−10, 7], *SD* = 18 ms, *t*(40) = −0.39, *p* = .699, *d*_*unb*_ = −0.08 [−0.51, 0.34].

**Fig. 8:**
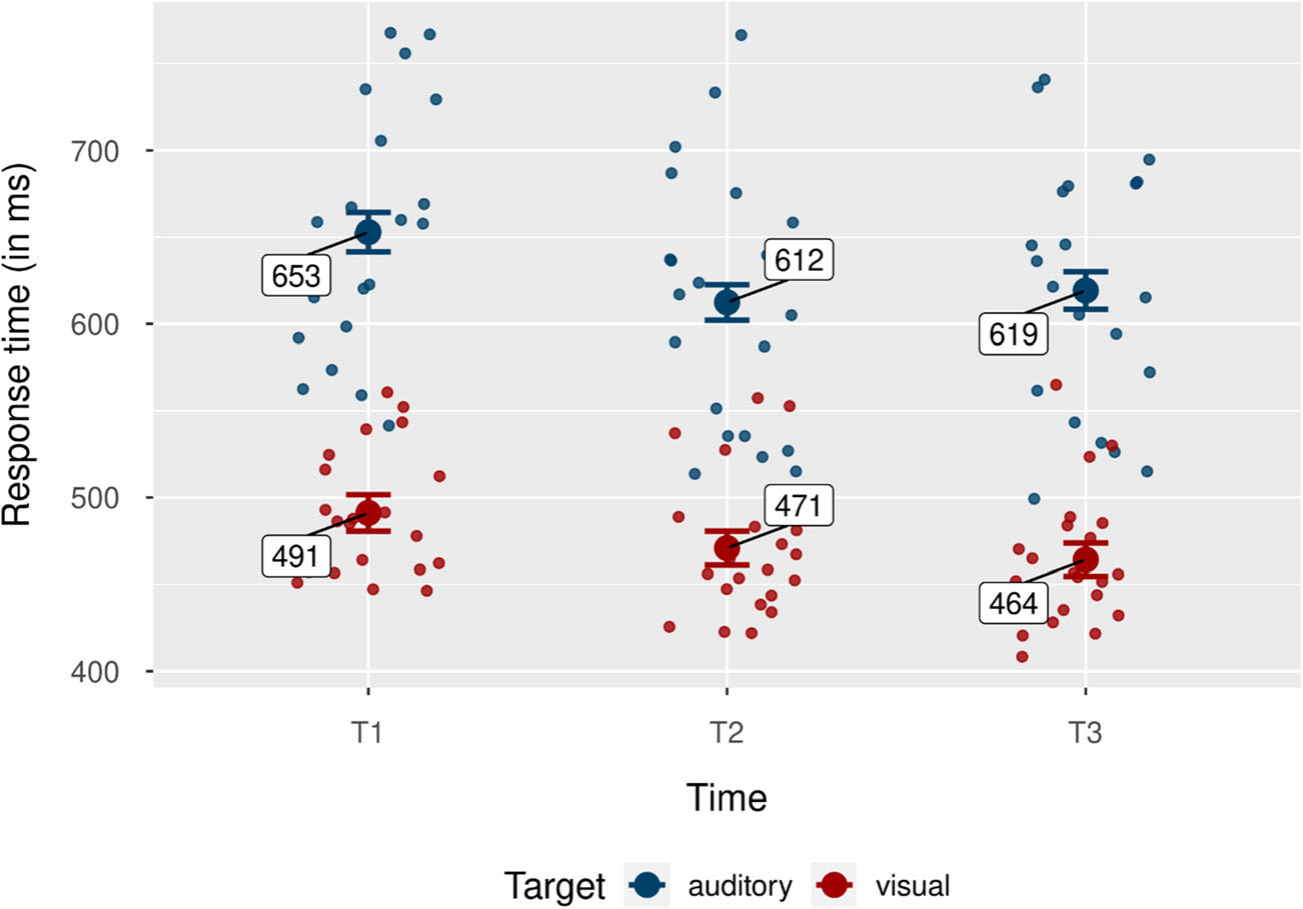
Response times by target modality for each time point. Blue dots indicate mean hit rates for the auditory target condition. Red dots show mean hit rates for the visual target condition. Individual data points represent mean hit rate per participant and condition. Error bars show 95% CI. (Colour figure online)

There was also a significant interaction between target modality and time, *F*(1, 20) = 4.16, *p* = .023, ${\upeta}_{\mathrm{p}}^2$ = .17. Figure 8 shows mean response times for each condition, together with individual means. Response times were faster for visual- compared with auditory targets at all three time points: T1, *M* = 162 ms, 95% CI [140, 184], *SD* = 48 ms, *t*(20) = 15.49, *p* < .001, *d*_*unb*_ = 3.25 [2.25, 4.50]; T2, *M* = 142 ms, 95% CI [118, 165], *SD* = 52 ms, *t*(20) = 12.37, *p* < .001, *d*_*unb*_ = 2.60 [1.76, 3.63]; T3, *M* = 155 ms, 95% CI [128, 182], *SD* = 59 ms, *t*(20) = 12.05, *p* < .001, *d*_*unb*_ = 2.53 [1.71, 3.54]. To further investigate this interaction, we computed difference values between visual and auditory RTs for each time point and compared them via *t* tests. The differences were larger at T1 compared with T2, *M* = 20 ms, 95% CI [7, 34], *SD* = 30 ms, *t*(20) = 3.11, *p* = .017, *d*_*unb*_ = 0.65 [0.20, 1.15]. Differences between T2 and T3 as well as between T1 and T3 were not significant, all *p*s > .092.

### Catch trial performance

**Fig. 9:**
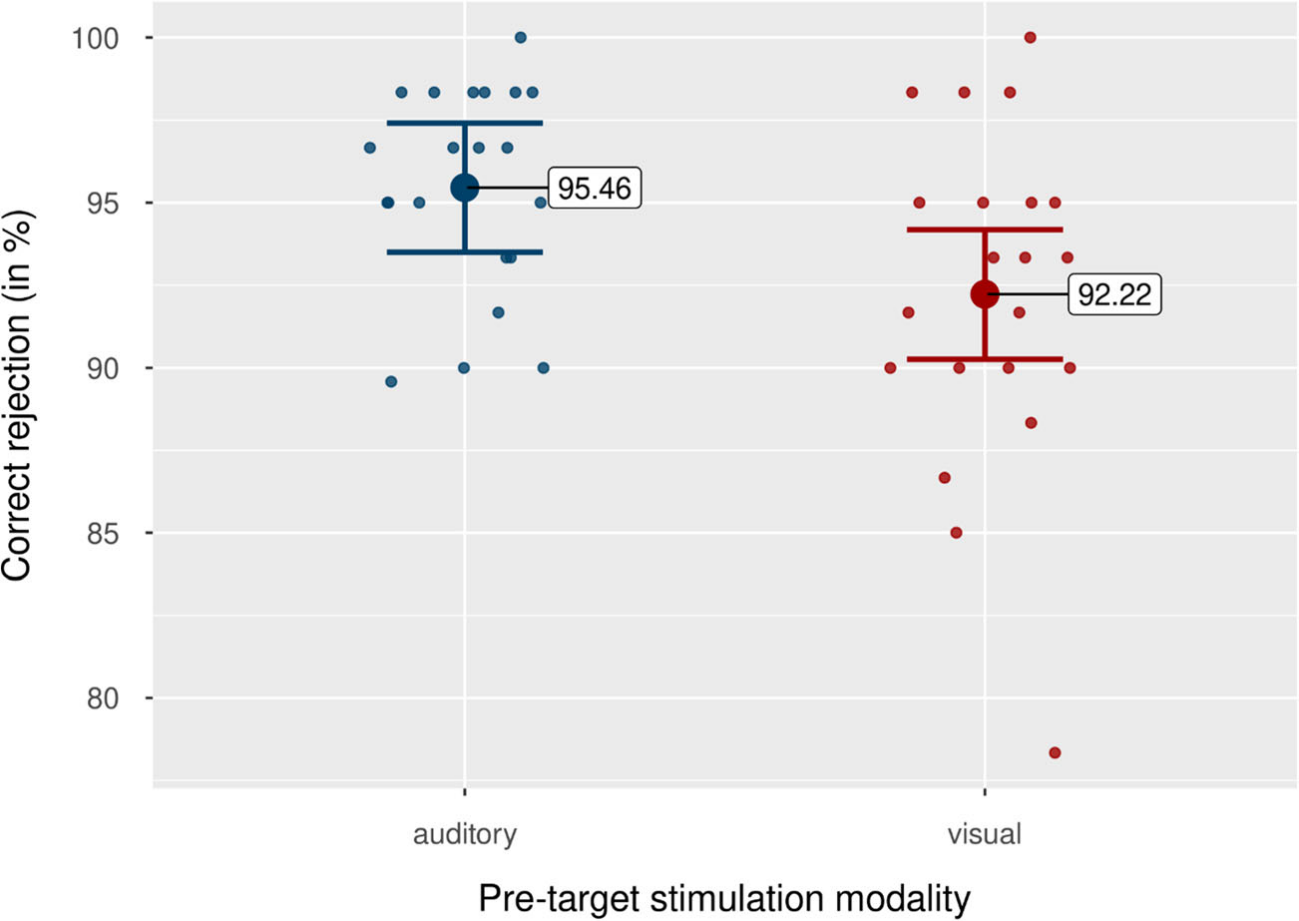
Catch trial performance by pretarget stimulation modality. Blue dots indicate correct rejections for trials with auditory pretarget stimulation, while red dots indicate trials with visual pretarget stimulation. Colored data points indicate mean correct rejections per subject. Error bars show 95% CI. (Colour figure online)

Figure 9 shows mean performance as a function of pretarget stimulation modality. Participants performed better in trials with auditory pretarget stimulation, *M* = 95.46%, 95% CI [93.50, 97.42], *SD* = 4.30%, than in trials with visual pretarget stimulation, *M* = 92.22%, 95% CI [90.26, 94.18], *SD* = 4.30%. A pairwise *t* test showed that this difference was significant, *M* = 3.54%, 95% CI [0.50, 6.60], *SD* = 6.70%, *t*(20) = 2.42, *p* = .025, *d*_*unb*_ = 0.51 [0.07, 0.98].

## Discussion

In the present study, we set out to investigate (1) whether the effects of rhythmic sensory stimulation can spread bi-directionally across sensory modalities, and (2) for how long such potential effects can last following rhythmic pretarget stimulation offset. We observed significantly better performance as well as more global, cross-modal effects following auditory versus visual pretarget stimulation. However, there was no evidence of cyclic performance modulation at the temporal rate of the sensory input, challenging the generality of phasic attentional modulations via rhythmic stimulation.

### No evidence of cyclic performance modulation

Contrary to our expectations, rhythmic sensory stimulation did not lead to convincing periodic effects in behavioural performance. While we observed significantly faster responses at in-phase compared with out-of-phase time points, the overall decrease in RTs over time (see Fig. 6) along with the interaction between phase and time suggests that this result reflects a more general trend. In other words, as Time Points 1, 3, and 5 were out-of-phase, and Time Points 2, 4, and 6 in-phase with the preceding stimulus, a linear trend in the performance can cause both a main effect of phase and an interaction between phase and time, in the absence of a genuine rhythmic fluctuation in performance. Notably, this finding is in line with the common variable foreperiod effect in ageing foreperiod distributions (Han & Proctor, 2022; Niemi & Näätänen, 1981), characterized by a negative slope for RTs as a function of foreperiod duration.

Similarly, the interaction between phase and time in hit rates can be explained by the inverted U-shaped trend in the data (see Fig. 4) rather than by a cyclic alternation between facilitated and impeded performance, as proposed by accounts of entrainment.

This negative finding is surprising, given that we designed our experiment closely to an influential study by Hickok et al. (2015), who reported rhythmic fluctuations in auditory target detection performance following rhythmic pretarget stimulation via 3 s of 3 Hz amplitude modulated noise. Arguably, however, their protocol differed in a number of important aspects from ours. First, they used auditory stimuli only, while our experiment employed both auditory and visual inputs for stimulation (varied between blocks) and targets (varied within blocks). Although we would have expected to replicate their findings at least for auditory targets in the auditory pretarget stimulation blocks, it is possible that participants were looking ahead, so that their prospective attention was divided between the visual and auditory modalities, thus, reducing the potential impact of auditory rhythmic pretarget stimulation on auditory target perception (MacDonald & Lavie, 2011; Molloy et al., 2015; Wahn & König, 2017). Second, while Hickok et al. employed a simple detection task, we presently used a target discrimination task. It might indeed be possible that rhythmic stimulation cyclically modulates specifically low-level sensory processing and the resulting mere detection threshold of stimuli in general, without additional changes to stimulus discriminability at later processing stages (e.g., via the tuning width of sensory networks; e.g., Demarchi et al., 2019; Zoefel & VanRullen, 2015). Finally, Hickok and colleagues (2015) presented targets at each quarter of the preceding stimulation period (i.e., at 0, 90, 180, 270 degrees), while we only presented them at the peaks and troughs (i.e., at 90 and 270 degrees). Although the sampling of additional time points might facilitate the detection of cyclic fluctuations, Hickok et al. observed a strong phase coherence across participants, with performance for all of them being in anti-phase with the rhythmic stimulus. Thus, at least in case of their data, sampling performance just at the peaks and troughs of the rhythmic stimulation cycle should have been sufficient to detect similar rhythmic modulations. Nevertheless, these points illustrate how entrainment effects could be more task-dependent than previously assumed, and potentially more pronounced for simple stimulus detection at early sensory processing stages. As further processing stages come into play for discrimination or bimodal tasks, those stages likely add noise to RTs and sensitivity measures. Diffusion models speak to this important distinction between tasks. These models take the neural noise during sensory processing, decision-making, and response generation into account (Dmochowski & Norcia, 2015; Hanes & Schall, 1996; Luce, 2008; MacDonald et al., 2006; Wood, 1977). The higher the extent to which a task taxes those stages, the more variance accumulates, impeding the detection of small behavioural effects.

In line with this reasoning, most of the previously successful demonstrations of sensory entrainment employed simple detection or unimodal discrimination tasks (e.g., Barnhart et al., 2018; de Graaf et al., 2013; Hickok et al., 2015; Lawrance et al., 2014; Miller et al., 2013; Saberi & Hickok, 2022; Spaak et al., 2014), with our present combination of a target discrimination task and two potential sensory modalities being complex and difficult in comparison

Importantly, while these differences could partly be responsible for our present lack of periodic behavioural effects, such an explanation would strongly argue against the generality and practical relevance of entrainment in everyday cognitive operations. Moreover, other recent studies have likewise failed to observe periodic behavioural effects in similar paradigms (de Graaf & Duecker, 2021; Duecker et al., 2021; Lin et al., 2021; Sun et al., 2021). For instance, Sun et al. (2021) performed an exact replication of the experiment by Hickok et al. (2015), and, like us, did not observe the proposed effects of rhythmic stimulation on auditory performance. Notably, however, they found the same inverted U-shaped pattern in hit rates as we did in our present study, thus, further corroborating our overall findings.

Lin et al. (2021), similar to our present study, employed an auditory pitch discrimination task, following slightly slower auditory stimulation at 2 Hz, 1.6 Hz, and 1.4 Hz. Testing a large sample of 181 participants in total, they likewise did not observe resulting cyclic performance modulations. Furthermore, Duecker et al. (2021) as well as de Graaf and Duecker (2021) reported no evidence of entrainment following rhythmic stimulation in the visual domain.

Thus, these recent studies, along with our present results, cast considerable doubt on the universality of cyclic performance modulation via rhythmic sensory stimulation (see also Keitel et al., 2022). Importantly, however, they do not pertain to the phenomenon of *ongoing* rhythmic fluctuations in performance (Fiebelkorn et al., 2018; Pomper & Ansorge, 2021; Re et al., 2019; VanRullen et al., 2014) which is likely dependent on neural oscillations as well. It is unclear, however, under which circumstances and to what degree these endogenous fluctuations can be synchronized with external rhythmic stimuli in a behaviourally relevant fashion.

### Auditory rhythmic stimulation boosts performance cross-modally

Interestingly, despite the lack of a temporally specific (i.e., cyclic) modulation of performance, we observed overall significantly faster RTs and higher hit rates following rhythmic auditory pretarget compared with visual pretarget stimulation. Along with significantly fewer false alarms in catch trials during auditory pretarget stimulation blocks, this suggests a genuine processing facilitation following auditory (rhythmic) stimulation, beyond a mere speed-accuracy trade-off. On top of that, there was an interaction between target and pretarget stimulation modality, indicating that auditory pretarget stimulation was equally affecting both auditory and visual target discrimination tasks, whereas the effects of visual pretarget stimulation were more confined to within the visual target modality (see Fig. 3).

A reason for the more pronounced effects following auditory pretarget stimulation might be, that naturally occurring rhythms in the presently used stimulation frequency of 3 Hz are more common and thus relevant in the auditory compared with the visual domain, particularly related to syllabic information rate in speech processing (Chandrasekaran et al., 2009; Elliott & Theunissen, 2009; Luc et al., 2012; Poeppel, 2003). Moreover, the propensity for sensory entrainment itself might in general be higher in the auditory compared with the visual domain, as auditory stimuli, unlike visual ones, by their very nature, unfold over time (Kubovy, 1988; VanRullen et al., 2014).

Our finding of a bimodal performance facilitation following auditory compared with visual pretarget stimulation would be in line with earlier reports of a cross-modal spread of entrainment effects from the auditory to the visual domain (Barnhart et al., 2018; Bauer et al., 2021). For instance, Bauer et al. (2021) found that a 3-Hz rhythmic auditory stimulus modulated visual target detection during (but not subsequent to) the stimulation. Using EEG, they also observed a concurrent entrainment of 3-Hz oscillatory neural activity over visual cortical areas. Interestingly, the study by Bauer et al. (2021) thus points to a potential mechanism underlying our present results: It is possible that rhythmic sensory stimuli, particularly auditory ones, induced increases in the amplitude of oscillatory neural activity, resulting in a locally enhanced 3-Hz (delta-theta band) activity. In other words, rhythmic stimulation could have had an effect on the *power* of neural activity, despite the lack of a behaviourally relevant *phase* effect*.* Such a mechanism would be in line with countless steady-state visual and auditory evoked potential studies, demonstrating clear spectral representations of rhythmic sensory input in electrophysiological signals (Ding et al., 2006; Norcia et al., 2015; Picton et al., 2003; Saupe et al., 2009).

In addition to genuine entrainment of neural activity, the rhythmicity of a sensory input in itself might be a salient feature that can draw attention to a stimulus. For instance, Stolte and Ansorge (2021) have recently shown that a visual cue flickering at a frequency of 5, 10, or 15 Hz draws spatial attention and facilitates the processing of subsequent targets at its location, compared with nonflickering cues. Ultimately, due to the purely behavioural nature of our experiment, we can only speculate whether our results reflect a genuine form of (phase-independent) neural entrainment or another process, potentially less dependent on the temporal regularity of the sensory input, and whether the proposed cross-modal link follows a similar route as suggested by Bauer et al. (2021).

A limitation of our present design is that due to the lack of a control condition (e.g., using an arrhythmic stimulation or a different stimulation frequency), it is unclear whether auditory stimulus facilitated or visual flicker impaired performance. As signals in the theta frequency (including the presently used 3 Hz) are naturally more common in the auditory than visual domain (Chandrasekaran et al., 2009; Elliott & Theunissen, 2009; Luc et al., 2012; Poeppel, 2003), and a highly visible flicker might be unpleasant for the observer, our results could also reflect reduced performance following rhythmic visual stimulation. However, if our rhythmic visual flicker indeed impaired performance, we would expect this impairment to be the same or even worse for subsequent visual targets (i.e., within the modality) than for auditory targets (i.e., across modalities). As we observed the opposite pattern, along with overall higher performance following auditory rhythmic stimulation (regardless of target modality), we believe our data reflect auditory facilitation rather than visual impairment. This interpretation is also in line with the recently observed facilitation of visual processing following 5-Hz stimulation (Stolte & Ansorge, 2021) discussed above. Regardless of the underlying mechanism, our data demonstrate that rhythmic sensory input can have immediate behavioural effects outlasting the stimulation itself, without a temporally specific, cyclic modulation of performance frequently associated with entrainment.

### Overall faster responses to visual targets

In addition to our main findings discussed above, we observed a pronounced processing advantage of visual versus auditory targets: Response times to visual targets were faster compared with auditory ones, especially at the first time point. Also, visual targets at the third time point yielded higher hit rates compared with auditory targets. This might seem surprising, as responses to auditory targets are commonly faster than to visual ones, at least in simple detection tasks (Jain et al., 2015; Laming, 1968; Shelton & Kumar, 2010; Welford, 1980). However, our current task required a discrimination and associated response between two target variants per modality. It is likely that the discrimination between visual flashes in the upper versus lower part of the target display was performed faster than the discrimination between high-pitched and low-pitched target sounds. Furthermore, there might have been a more natural spatial congruency between the responses via the up- and down arrow keys and the upper and lower visual targets, respectively, compared with the high-pitched and low-pitched sounds, respectively. Additionally, only auditory but not visual targets had to be categorized from memory (i.e., matched with the initially learned representation of the high or the low tone). Finally, since we dynamically adjusted target intensity to yield an average hit rate of about 75%, it is likely that, if auditory target performance was more difficult than visual performance (e.g., due to memory requirements not present in the visual task), auditory performance was subject to a more conservative response threshold (or a speed–accuracy trade-off), resulting in significantly slower response times along with similar hit rates compared with visual target performance.

## Conclusions

We set out to investigate the potential presence and duration of auditory pretarget and visual rhythmic pretarget stimulation effects on target processing, both within and across modalities. Unexpectedly, but in line with several recent studies and the category of discovery-oriented research, we observed no evidence for cyclic fluctuations in behavioural performance following rhythmic sensory stimulation. This was despite the fact that our task design closely resembled that used in previous successful demonstrations of sensory entrainment. However, our data demonstrated a more general, temporally less specific effect of rhythmic sensory stimulation, in the form of within- and cross-modally facilitated behavioural performance following auditory compared with visual stimulation. In addition to a potentially higher salience of auditory rhythms, this could indicate an effect on oscillatory 3-Hz power, resulting in facilitated cognitive control and attention. Taken together, our study further challenges the generality of periodic behavioural modulation via rhythmic sensory stimulation, while demonstrating a modality-independent attention effect following auditory rhythmic stimulation.

## Footnotes

1 Here, we use a fairly broad definition of the term ‘cross-modal,’ referring to any case in which a manipulation of one sensory modality leads to subsequent behavioural consequences in another modality.

2 Please not that given the purely behavioural nature of our experiment, we subsequently use the term ‘rhythmic stimulation’ rather than ‘entrainment,’ as we cannot say whether our protocol leads to actual entrainment on a neural level.
